# Conference Calendar

**DOI:** 10.1002/cfg.143

**Published:** 2002-02


					Conference Calendar
March-May 2002
Animal and plant models
Plant Genetic Resources in the Genomic Era:
Genetic Diversity, Genome Evolution and
New Applications (6th Gatersleben Research
Conference) - March 7-11
http://www.ipk-gatersleben.de/meetings/ipk6grc.htm
3rd International Amsterdam Mouse
Symposium - March 11-13
http://www.mousephysio.com/ams3.html
43rd Annual Drosophila Research Conference
- April 10-14
http://www.drosophila-conf.org/
Bacteria
150th Society for General Microbiology
Ordinary Meeting - April 8-12
http://www.socgenmicrobiol.org.uk/MTGPAGES/
warwick02.htm
102nd ASM General Meeting - May 19-23
http://www.asmusa.org/mtgsrc/generalmeeting.htm
Bioinformatics
SAC2002: 17th ACM Symposium on Applied
Computing, Bioinformatics Track - March
10-14
http://www.cs.msstate.edu/ybioin/
SocBiN - Bioinformatics 2002: 4th
Scandinavian Bioinformatics Conference -
April 4-7
http://www.ii.uib.no/bio2002/
Evolution/comparative genomics
The Genetics Society/British Society for
Developmental Biology - The Evolution of
Developmental Mechanisms - March 20-23
http://www.genetics.org.uk/
General genomics
ABRF Biomolecular Technologies (Proteomics
and Genomics) - March 9-12
www.faseb.org/meetings/abrf2002
DNA in Chromatin: At the Frontiers of
Biology, Biophysics and Genomics - March
23-29
http://adn.crpp.u-bordeaux.fr
Society for Experimental Biology - Annual
Main Meeting - April 8-12
http://www.sebiology.org/meetings/2002/Swansea/
index.htm
FASEB - Experimental Biology: Genome -
April 20-24
http://www.faseb.org/meetings/eb2002/call/
CSHL - Genome Sequencing and Biology -
May 7-11
http://nucleus.cshl.org/meetings/2002genome.htm
ESHG - European Human Genetics
Conference - May 25-28
http://www.eshg.org/strasbourg_2002.htm
Metabolomics
1st International Conference on Plant
Metabolomics - April 7-11
http://www.metabolomics.nl/
Comparative and Functional Genomics
Comp Funct Genom 2002; 3: 79-80.
DOI: 10.1002/cfg.143
Copyright # 2002 John Wiley & Sons, Ltd.
Pharmacogenomics
HPCE 2002: 15th International Symposium on
Microscale Techniques - April 13-18
http://www.chemsoc.se/sidor/KK/hpce2002/hpce2002.htm
IBC - Drug Discovery Technology 2002 -
April 15-18
http://www.drugdisc.com/stuttgart/
MipTec/ICAR 2002: 4th International
conference on microplate technology,
laboratory automation and robotics - May
27-30
http://www.messebasel.ch/miptec/
Proteomics
SRI - Proteomics: Protein Discovery to
Validated Drug Targets - March 13-14
http://www.srinstitute.com/part_iter_site_page.cfm?
iteration_id=272
CHI - Proteins to Profits: Proteomics Europe
& Protein and Peptide Arrays - March 25-27
http://www.healthtech.com/2002/pre/index.htm
http://www.healthtech.com/2002/pce/index.htm
IBC - Protein Microarrays - March 18-22
http://www.lifesciencesinfo.com/2712
IBC - Proteomics: Delivering New Routes to
Drug Discovery - May 6-9
http://www.lifesciencesinfo.com/2702
Structural genomics
9th International Conference on the
Crystallisation of Biological Macromolecules
(ICCBM9) - March 23-28
http://www.conventus.de/iccbm9
Transcriptomics
IBC - Emerging Microarray Technologies and
Applications - March 18-22
http://www.lifesciencesinfo.com/2712/
Yeasts and fungi
2nd International Fission Yeast Meeting -
March 25-30
http://www.bio.sci.osaka-u.ac.jp/pombe2002/
6th European Congress on Fungal Genetics -
April 6-9
http://www.agr.unipi.it/ECFG6/
150th Society for General Microbiology
Ordinary Meeting - April 8-12
http://www.socgenmicrobiol.org.uk/MTGPAGES/
warwick02.htm
102nd ASM General Meeting - May 19-23
http://www.asmusa.org/mtgsrc/
gm2002prelimprogtoppage.htm
Key
ASM - American Society for Microbiology [http://
www.asmusa.org]
CHI - Cambridge Healthtech Institute [http://
www.healthtech.com]
CSHL - Cold Spring Harbor Laboratory [http://
www.cshl.org/]
ESHG - European Society of Human Genetics
[http://www.eshg.org]
FASEB - Federation of American Societies for
Experimental Biology [http://www.faseb.org]
IBC - IBC conferences [http://www.ibcusa.com]
SocBiN - Society for Bioinformatics in the Nordic
Countries [http://www.socbin.org/]
SRI - Strategic Research Institute [http://www.
srinstitute.com]
The Conference Calendar is a listing of forthcoming conferences of interest to our readership. We provide
the title, dates and web address of each conference, to enable readers to find out more information.
Inclusion does not imply endorsement by the Journal. If you know of a relevant conference, please contact
the Managing Editor.
80 Conference Calendar
Copyright # 2002 John Wiley & Sons, Ltd. Comp Funct Genom 2002; 3: 79-80.

				

